# Assessing the Efficacy of Alkylating Agent Regimens in the Treatment of Infantile Malignant Osteopetrosis: Cyclophosphamide, Busulfan, or Thiotepa

**DOI:** 10.7759/cureus.26600

**Published:** 2022-07-06

**Authors:** Himanshu Wagh, Amber Arif, Akshay J Reddy, Ethan Tabaie, Aditya Shekhar, Mildred Min, Neel Nawathey, Mark Bachir, Hetal Brahmbhatt

**Affiliations:** 1 Medicine, California Northstate University College of Medicine, Elk Grove, USA; 2 Ophthalmology, California University of Science and Medicine, Colton, USA; 3 Neurobiology, Physiology, and Behavior, University of California (UC), Davis, USA; 4 Health Sciences, California Northstate University, Rancho Cordova, USA; 5 Psychiatry, Mercy General Hospital, Sacramento, USA

**Keywords:** meta-analysis, infantile, graft vs host disease, treatment choices, alkylating agents, hematopoiesis, thiotepa, busulfan, cyclophosphamide, osteopetrosis

## Abstract

Infantile malignant osteopetrosis is a debilitating disease that requires total bone marrow irradiation and transplant procedures for patients to survive. The major complication of this procedure is graft vs host disease (GVHD), followed by infections and end organ toxicity. Therefore, current research efforts into treatment mainly aim to reduce GVHD while limiting infections and organ toxicity. Different regimens of alkylating agents have been used to try to reduce GVHD. The most common regimen is cyclophosphamide (Cy) with busulfan (Bu), followed by Cy with Bu and thiotepa (Thio). This meta-analysis aimed to evaluate the efficacy of different treatments by comparing mortality and morbidity causes and rates across groups. The mean one-year survival rate for the Cy, Bu, Thio regimen studies in the human leukocyte antigen (HLA) unmatched group (45.01%) was statistically lower than the one-year survival rate for the studies using just a Cy, Bu regimen (70.8%) in the HLA unmatched studies (p<0.00142). The one-year survival in the studies which had HLA-matched donors was 80.56%, which is statistically higher (p<0.001) than the one-year survival in the HLA-unmatched studies (53.96%), indicating a benefit of finding HLA-matched donors. It seems that price and availability could be a factor in the widespread use of Cy.

## Introduction and background

Osteopetrosis, also known as marble bone disease, is known as a rare skeletal disorder that leads to increased bone density and fragility. There are multiple forms of osteopetrosis based on genetic inheritance-autosomal dominant and recessive. The autosomal recessive form is also known as infantile malignant osteopetrosis (IMO) and presents with varying symptoms based on the mutation [1]. The most common symptoms that a patient will present include frontal bossing, depressed nasal bridge, mandible protrusions, spontaneous fractures of ribs and long bones, vision and hearing loss, and short stature. On an x-ray, the patient will have an increase in opacification of the bones. It can also be diagnosed before birth; at 11-14 weeks of gestation, osteopetrosis can be diagnosed through amniocentesis and chorionic villus sampling [2].

This disease is due to dysfunction of osteoclasts, which are cells specialized in the breaking down and absorption of bone, leading to an increase in bone mass. This will lead to an accumulation of bone mass with abnormal architecture composed of bony overgrowth, making the bones more susceptible to fractures. The two different classifications of osteopetrosis are based on genetic inheritance - autosomal dominant and autosomal recessive. The autosomal dominant form, also known as Albers-Schönberg disease, is generally asymptomatic and typically discovered as an incidental finding on x-ray. The autosomal recessive form, also known as infantile malignant osteoporosis, is more fatal. Osteopetrosis can be due to a mutation in the carbonic anhydrase 2 gene, which results in deficient hydrogen ion pumping and thus decreases the ability of osteoclasts to make an acidic environment for bone resorption. Other known genes involved with osteopetrosis include CLCN7, OSTM1, TCIRG1, TNFSF11, PLEKHM1, and TNFRSF11A [3]. 

Infantile malignant osteopetrosis is diagnosed in 8-40 children annually within their first year in the United States, with an average incidence of 1:200,000 [1]. Without proper diagnosis and treatment, the average life expectancy of most children is two to ten years depending on complications such as infections or bone marrow failure. Other complications include hepatosplenomegaly with anemia, frontal bossing with macrocephaly, exophthalmos, and failure to thrive. Affected individuals also present with low calcium and parathyroid hormone (PTH) levels. Even with treatment, the majority of children die in the first decade of life [2].

Although there are no methods to prevent osteopetrosis, there are a few treatment options presented to the patient that vary upon severity of the disease and status of the patient. Vitamin D and Ca supplements are normally offered to the patient to stimulate osteoclasts, which can help decrease or reverse complications of osteopetrosis. Interferon gamma-1b is also a known treatment that uses hematopoiesis to increase bone resorption and drastically improve leukocyte function long term [3].

One of the most used options is intravenous hematopoietic stem cell transplants (HCT) to increase bone resorption with these infused osteoclasts. For this method to work, the host’s bone marrow must be completely irradiated. This is done with radiation and alkylating agents. The most common regimen of alkylating agents is cyclophosphamide with busulfan, although many other regimens are available and are used often. After the irradiation, bone marrow from a human leukocyte antigen (HLA)-matched donor must be given so that it can proliferate and eventually fill the host’s marrow space. This functional bone marrow will be able to produce functional osteoclasts [4]. 

Even though this treatment plan is widely used, one of the major concerns of this treatment is immunosuppression. With a weak immune system, patients are more susceptible to infections, diseases, and cancer. Another danger that claims the lives of over 10% of HCT patients is graft vs host disease (GVHD), a reaction between residual host cells and transplanted cells [5,6]. There are many factors that must be considered for this treatment plan to be successful and to cure infants from IMO. This study will explore various alkylating agent regimens and assess their relative efficacy in terms of one-year survival rate, as well as explore various other factors that could lead to better outcomes such as HLA matching, dose adjustment, and coadministration of other drugs to prevent complications.

## Review

Methods

**Figure 1:**
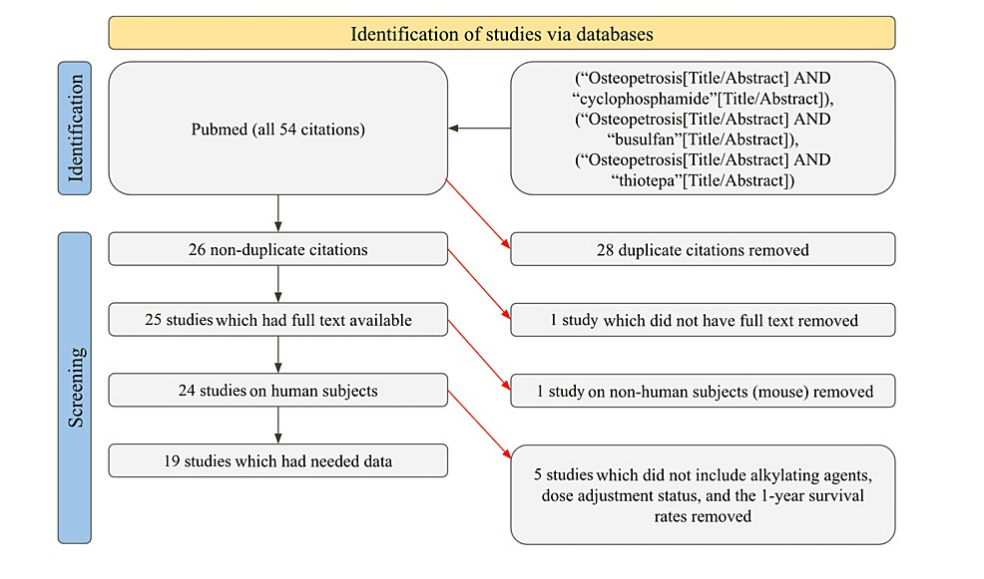
PRISMA diagram on study identification PRISMA: Preferred Reporting Items for Systematic Reviews and Meta-Analyses

Searches were conducted on PubMed to find studies about the use of alkylating agents for the treatment of malignant infantile osteopetrosis. Exact searches were done with the keywords “osteopetrosis and cyclophosphamide,” “osteopetrosis and busulfan,” and “osteopetrosis and thiotepa.” All time frames were searched. This brought a pool of 54 studies, of which 26 were not duplicates, 25 had full text available, 24 were human studies, and only 19 had the information needed for the tables. The data collected from these 19 studies included the alkylating agents used for preparative conditioning, whether the doses were weight adjusted, and the one-year survival rates for the patients in the studies. Studies that did not report these results were excluded. Studies that focused on other animals such as mice were excluded. In order to remove personal bias, no studies which fit the criteria were excluded. The filtering process that was utilized by the authors for this investigation can be seen more clearly in Figure 1.

All data were retrieved from studies with proper patient consent. No personal information such as names or ages of participants was included in this review, and only values of one-year survival rate were included to avoid breaches of privacy and to remain ethical.

In order to compare the impact of HLA matching, studies were separated into two categories - HLA matched and HLA unmatched. The data for these categories were placed in Table 1 and Table 2, respectively. HLA matched was defined as a ⅚ match or more. Averages for the one-year survival rate for these two categories were found and compared using a one-tailed t-test to see if the differences in means were significant with a 95% confidence interval. These results were reported in Table 3. Additionally, for Table 1, one-year survival rates were compared with a one-tailed t-test with a 95% confidence interval between studies that used cyclophosphamide and busulfan and studies which used other regimens.

**Table 1:** HLA-unmatched studies HLA: human leukocyte antigen; Cy: cyclophosphamide; Bu: busulfan; Thio: thiotepa; Mel: melphalan

Author (year)	Alkylating agent	Dose adjustment	1-year survival
Schulz et al. (2002) [7]	Cy, Bu, Thio	y	4/6
Even-Or et al. (2021) [8]	Cy, Bu, Thio	y	3/5
Orchard et al. (2015) [9]	Cy, Bu, Thio	n	34/79
Llano et al. (2008) [10]	Cy, Mel	y	0/1
Top 4	N/A	N/A	41/91 = 45.01%
Al-Seraihy et al. (2021) [11]	Cy, Bu	y	5/6
Sieff et al. (1983) [12]	Cy, Bu	y	1/1
Fischer et al. (1986) [13]	Cy, Bu	y	1/2
Jaing et al. (2006) [14]	Cy, Bu	y	1/1
Bahr et al. (2016) [15]	Cy, Bu	y	1/3
Zhu et al. (2021) [5]	Cy, Bu	y	20/27
Behfar et al. (2015) [16]	Cy, Bu	y	2/4
Slatter et al. (2004) [17]	Cy, Bu	n	1/1
Zhu et al. (2012) [18]	Cy, Bu	n	2/3
Cy, Bu studies	N/A	N/A	34/48 = 70.8%
Total average	N/A	N/A	75/139 = 53.96%

**Table 2:** HLA-matched studies HLA: human leukocyte antigen; Cy: cyclophosphamide; Bu: busulfan; Thio: thiotepa

Author (year)	Alkylating agent	Dose adjustment	1-year survival
Torres et al. (2021) [19]	Cy, Bu, Thio	y	1/2
Rosales et al. (1999) [20]	Cy, Bu, Thio	y	3/4
Sieff et al. (1983) [12]	Cy, Bu	y	1/1
Solh et al. (1995) [21]	Cy, Bu	y	4/8
Othman et al. (2009) [22]	Cy, Bu	y	1/1
Behfar et al. (2015) [16]	Cy, Bu	y	14/15
Neven et al. (2019) [23]	Cy, Bu	y	4/4
Coccia et al. (1980) [24]	Cy	y	1/1
Average	N/A	N/A	29/36 = 80.56%

**Table 3:** Statistical significance between group means HLA: human leukocyte antigen; Cy: cyclophosphamide; Bu: busulfan

Group comparison	t-Value	p-Value
HLA-matched vs unmatched	11.999	<0.0001>
HLA-unmatched Cy+Bu vs HLA-unmatched other	3.05866	0.00142

Results

The HLA-matched category (n=36) had a one-year survival rate of 80.56%, while the unmatched category (n=139) had a survival rate of 53.96%. The one-tailed t-test yielded a p-value of <0.0001, indicating a significant difference between the HLA-matched and HLA-unmatched groups.

Studies in the HLA-unmatched category used different alkylating agent regimens. Therefore, the most common regimen, Cyclophosphamide with Busulfan, was compared within the four studies which used other regimens. Cyclophosphamide with Busulfan (n=48) had an average one-year survival rate of 70.8% while the other regimen category (n=91) had an average one-year survival rate of 45.01%. The other regimen category consisted of three studies including Cy, Bu, Thio, and one study including Cy and melphalan (mel). A one-tailed t-test yielded a p-value of 0.00142, which is less than 0.05, indicating that the means are significantly different.

Discussion

Malignant infantile osteopetrosis is a debilitating disease and is inherently related to the immune system. Overgrowth of bone tissue disrupts the bone marrow, causing extramedullary hematopoiesis and immunodeficiency. Thus, it is paramount to diagnose and treat the disease as soon as it is detected. The procedure to cure osteopetrosis, however, is not void of flaws and risks. Since osteopetrosis is a defect in the formation of functional and productive osteoclasts, the disease is treated with HCT to introduce healthy osteoclasts to the body. However, HCT is risky, as it leads to a high incidence of GVHD. Indications for HCT include bone marrow failure and age of less than one year HLA matching is one of the most important considerations for reducing GVHD rates and mortality [3]. However, completely matched HLA donors are often not available, necessitating the use of heavier immunosuppression through alkylating agents. The dosage and type of immunosuppressive agent regimen could affect mortality rates due to their properties. In addition, the use of dose adjustment is beneficial to provide the right levels of immunosuppression. If excessive immunosuppression is administered, infection incidence increases, for which several strategies exist to reduce morbidity and mortality. Beyond this, the availability and price of the medications dictates which alkylating agents are most often used. However, it remains difficult to arrive at concrete conclusions due to the many confounding variables in the different studies included in this analysis.

There are different causes of death for IMO patients undergoing therapy, including insufficient hematopoiesis, infection, and GVHD. If there is insufficient immunosuppression, residual host cells attack the transplanted cells, and the transplanted cells launch an immunological response as well. This occurs because the cells from both the “graft” or transplant, and the “host” recognize each other as non-self because their HLA proteins are different. The main effector cells for GVHD are graft T cells, which recognize any of the host’s cells as foreign and rapidly proliferate to cause systemic damage in a type 4 hypersensitivity reaction. Symptoms can be seen in the skin, gastrointestinal tract, and liver. In the skin, there is often a maculopapular rash that spans across the palms, soles, shoulders, and the nape of the neck. Abdominal pain and diarrhea are also common, for which blood transfusions are sometimes necessary to restore fluid balance. Liver damage is indicated by elevated bilirubin and alkaline phosphatase levels. These signs must be very closely monitored to diagnose GVHD so that it can be treated fast with more immunosuppression. If it is not treated fast, it can progress rapidly and lead to death [6,24].

In the studies cited in Tables 1, 2, GVHD was one of the main causes of death. It affected the HLA-unmatched group more. This result is expected because higher numbers of matched HLA proteins lead to higher incidences of the bone marrow cells recognizing each other as self-cells and not initiating a hypersensitivity reaction. HLA matching significantly reduces death rate by reducing GVHD-caused deaths, confirmed by the average one-year survival rate for the HLA-matched group being 80.56% while the one-year survival rate for the HLA-unmatched group being 53.96% [25]. Other sources have also come to the same conclusion that HLA matching is paramount in transplant-related surgeries. The consensus opinion of the Blood and Marrow Transplant Clinical Trials Network (BMT CTN) is that the donor and recipient should be a 6/6 match at HLA-A, -B, and -DRB1. Siblings of the patient will have the highest chances of having matching HLA alleles. If a sibling is not available, as it is in ⅔ of cases in the United States, then a parent donor is the next best option [26,27]. Although it is more unlikely to get a good match from a parent, that is what most patients have to do.

Immunosuppressive drugs are diverse, and all have their own indications and toxic side effects. The class that is most often used in BMT are alkylating agents, which work by cross-linking strands of DNA, leading to loss of functionality and apoptosis. Cyclophosphamide, busulfan, and thiotepa are the three main drugs used in the studies included in Tables 1, 2. Melphalan was only used in the study conducted by Llano et al. [10].

Cyclophosphamide is a nitrogen mustard alkylating agent, which permanently binds to DNA within and between adjacent DNA strands at the guanine N-7 position, leading to apoptosis. The drug is metabolized by cytochrome P-450 to produce hydroxycyclophosphamide, which is then metabolized again to aldophosphamide, which then splits to form phosphoramide mustard and acrolein, of which phosphoramide serves the alkylating function and acrolein is toxic and leads to hemorrhagic cystitis. Because of the common side effect of hemorrhagic cystitis, patients on cyclophosphamide are encouraged to drink a lot of water and stay hydrated [28].

Busulfan, another alkylating agent, was found to be the second most used in the literature. Its mechanism of action is similar to that of cyclophosphamide in that it substitutes alkyl groups for hydrogen atoms. The molecule has methanesulfonate groups located on opposite ends of a four-carbon chain. A hydrolysis reaction happens between these two groups and two highly reactive carbonium cations are released, which then undergo a nucleophilic substitution reaction to replace guanine molecules, creating cross links [29]. Although Bu leads to the common side effects of all alkylating agents (intestinal mucosal damage, alopecia, pancytopenia, anemia, amenorrhea, impaired spermatogenesis, and increased risk of malignancy), it also has its own unique side effects [29]. These include hepatic veno-occlusive disease, interstitial pulmonary fibrosis, busulfan-induced seizure, hyperpigmentation, emesis, wasting syndrome, thrombocytopenia, and sometimes medullary aplasia [29].

Thiotepa is believed to function the same as other alkylating agents. It also shares most of its adverse effects with the other alkylating agents. The main adverse effect of Thio is hepatotoxicity, which arises as a direct cytotoxic injury to rapidly dividing cells [30]. Melphalan, which was used in one study, is a phenylalanine derivative of nitrogen mustard that forms a variety of DNA adducts and crosslinks. Melphalan induces chromosomal aberrations, sister chromatid exchange, micronuclei, and mutations in DNA, leading to the development of post-op acute myeloid leukemia or other cancers [31]. This toxicity may be a reason for the low use of the drug.

Because mortality can result from both insufficient and excessive immunosuppression, it is paramount to find the optimal balance. Dosing is an important variable that needs to be explored more. The studies used different values of alkylating agent mg/L to be given to the patients. If the regimen included three alkylating agents, the doses were less. But even among those, there was variation. Due to this complexity, the variable of dosing was not compared in this review. Future research could be done to explore which dosing regimen yields the most benefit. Such a study could explore how the causes of death and one-year survival change in incidence as the doses are adjusted slightly from the commonly implemented regimens. Controlling for regimen and other factors such as age will yield valuable data on the ideal dosage.

Different regimens have been used, and cyclophosphamide with busulfan has become the norm. Nine out of 13 studies for HLA-unmatched category used Cy+Bu, while five out of eight studies for the HLA-matched category used Cy+Bu. For the HLA-unmatched category, it was found that the Cy+Bu regimen studies led to significantly higher one-year survival rates compared to the studies which used other regimens. A major contributing factor to this difference was the study by Orchard et al., which had 79 participants and only 34 of them survived after 1-year [9]. This was a big outlier in the other regimen category in Table 1. In Orchard et al.'s study, the main cause of death was GVHD, accounting for 43% of deaths in the HLA-unmatched group. In this group, interstitial pneumocystis accounted for 10% of deaths [8]. Veno-occlusive disease occurred in 44% compared to 12.5% in the HLA-matched group. Ten patients received reduced intensity conditioning regimens, and only three of those patients survived. This study differed from others in that it spanned two decades, so the earlier treatments did not use common practices such as dose adjustment. The study strongly encourages the use of dose adjustment, particularly for busulfan to “improve engraftment and lower end organ toxicity, particularly veno-occlusive disease” [9].

There exists literature comparing different regimens. As for Cy+Bu vs Cy+TBI, the results are contrariant. A study comparing regimens for stem cell transplants in chronic myeloid leukemia (CML) patients found that Cy with Bu had the same therapeutic efficacy as Cy with total body irradiation (TBI), so the choice of one regimen over the other is due to other factors [32]. Another study comparing Cy with TBI and Cy with Bu for hematopoietic stem cell transplants for leukemia patients found that Cy+TBI had significantly less transplant-related mortality [33]. These results are the products of different studies which both had different conditions, so it is difficult to arrive at a conclusion.

For the HLA-unmatched group, the main alternative regimen to Cy+Bu was Cy+Bu+Thio. Compared these two regimens for bone marrow transplants in thalassemia patients and found no significant differences in patient outcomes [34]. The study conducted by Rosales et al. aimed to evaluate the effect of the addition of Thio to the classical Cy+Bu regimen [20]. It found that overall rates of toxicity were lower in the group with Thio added. The two main complications were mucositis and gastrointestinal toxicity in Rosales et al.'s study [20]. A different study found Thio in doses exceeding 1 g/m^2^ led to higher rates of hepatotoxicity [35]. Although Thio can lead to liver failure, this study found that the number of renal replacement therapy operations in the group with Thio was similar to the group without Thio. The data suggest a dose-dependent relationship, for which lower doses yield statistically similar hepatic side effects to other alkylating agents. The benefits of adding Thio to the regimen were unclear in this study, and the findings reported that only a randomized study with a bigger sample size could assess the superiority of a particular regimen.

Another possible factor contributing to the differences in one-year survival rates is whether the drugs were dose adjusted for the weight of the patient. Administration of standard amounts of drugs would not lead to the same systemic drug concentrations in patients of different weights. So, dose adjustment status was noted down for each study to investigate whether this variable had an impact on mortality. There were three studies that did not have dose adjustment or did not report dose adjustment, of which Orchard et al.'s study was the biggest. Orchard et al.'s study had a one-year survival rate of 34/79, or 43%, which is lower than the mean for the HLA-unmatched group (45.01%). The lack of dose matching may have been a reason for the low survival rate. The literature has proven dose adjustment to be important in reducing mortality [36]. Thus, dose adjustment is a common practice in BMT.

Another big cause of death for patients of all HLA statuses was infection. Opportunistic infections such as Aspergillus, *Pneumocystis jirovecii*, Cytomegalovirus, herpes zoster, Epstein-Barr, tuberculosis, and others are common infections post-transplant [37]. These should be closely monitored, and the patient must receive prompt treatment in order to prevent death from these causes. Too much immunosuppression leads to higher rates of infection and resulting mortality [38]. Thus, a careful balance is needed, for which considerable research must be done and many variables must be considered.

Literature exploring avenues of reducing infection-related deaths in bone marrow transplant patients exists. Isolation in laminar air flow rooms results in reduced infectious deaths and delayed contraction of a fatal infection [39]. However, rooms like these are often not available in hospitals. Total gut decontamination has also been considered, and it was found that selective decontamination has not led to fewer febrile episodes or to lower mortality in neutropenic patients [40]. The use of granulocyte colony stimulating factor has shown some benefits as well. Another investigation found two years that the post-transplant survival rate was 39% in patients with recombinant human granulocyte colony-stimulating factor (rhG-CSF) and 24% in patients without rhG-CSF [41]. The only drawback is that rhG-CSF led to higher incidence of GVHD. Another promising route is the use of prophylactic antibiotics to reduce infection-related deaths, and the results show pros and cons of the practice. The study conducted by Horton et al. found that prophylactic antibiotics do reduce the incidence of mortality from infections, but they also lead to toxic side effects, higher risk of antibiotic resistance, more damage to the gut microbiota, and potential *Clostridium difficile* infection [39]. Due to these drawbacks, prophylactic antibiotics should be administered based on patient characteristics, neutropenia degree, and comorbidities.

Another variable that needs to be highly taken into consideration is pricing and availability. Fortunately, busulfan does have multiple generic forms, which allows for lower costs of this drug. Drugs.com lists its price at $8.71/mg. The same website lists thiotepa at $27.8/mg. The higher cost of thiotepa could be a reason most regimens did not add Thio and administered Cy with Bu instead. Cyclophosphamide is available for $0.00231/mg and is the cheapest alkylating agent available [42]. This may be a factor that has led to its widespread use.

One source of possible error is that studies often administered drugs other than Cy, Bu, and Thio to the patients. Commonly administered drugs other than the aforementioned ones include fludarabine, rituximab, and alemtuzumab. As these other drugs were not alkylating agents, they were not studied in this study. This diverse array of drugs available increases the complexity of comparing regimens. Each of these drugs was administered at different doses in each study, further adding confounding variables to the already difficult comparison between alkylating agents. Beyond maximizing efficacy, factors such as comorbidities influence which drugs can be given to prevent adverse side effects, which is why different drugs were used on different patients from the same study. More research also needs to be done into the benefits of the different regimens. Such data will lead to better procedures and hopefully better outcomes for the patients diagnosed with IMO.

## Conclusions

Infantile malignant osteopetrosis is a debilitating disorder that has to be treated early with total body irradiation and complete bone marrow transplant. Different alkylating agent regimens are used to irradiate the host’s bone marrow, including cyclophosphamide, busulfan, thiotepa, and fludarabine, of which Cy+Bu was found to lead to the statistically highest one-year survival rate of 70.8% in HLA-unmatched group. Dose adjustment was found to be crucial practice as it minimizes the chance of severe immunosuppression and infection from drug over-administration and minimizes the chance of GVHD from drug under-administration. HLA matching was found to lead to higher one-year survival rates of 80.56% compared to 53.96% in HLA-unmatched individuals. Future research should focus on the impact of other factors such as co-administration of other non-alkylating agent immunosuppressants, antimicrobial drugs, and rhG-CSF, and how these affect the quality of treatment.
